# OPRM1/MRGPRX1 heterodimers drive opioid-induced itch through a peripheral mechanism

**DOI:** 10.1186/s12929-026-01238-x

**Published:** 2026-03-28

**Authors:** Babina Sanjel, Diwas Rawal, Myeong Ryeo Kim, Wook-Joo Lee, Kwang Won Jeong, Won-Sik Shim

**Affiliations:** 1 College of Pharmacy, Gachon University, Hambangmoe-ro 191, Yeonsu-gu, Incheon, 21936 Republic of Korea; 2 Gachon Institute of Pharmaceutical Sciences, Hambangmoe-ro 191, Yeonsu-gu, Incheon, 21936 Republic of Korea

**Keywords:** Opioid, Heterodimer, OPRM1, MRGPRX1, Pruritus, Peripheral sensory neuron

## Abstract

**Background:**

Opioid-induced itch is a common and distressing side effect of opioid analgesics, yet its underlying mechanisms remain poorly understood. While central µ-opioid receptor (OPRM1) signaling has been implicated, emerging evidence suggests that peripheral mechanisms also contribute, although their specific roles have not been clearly defined.

**Methods:**

We investigated the interaction between OPRM1 and the itch-specific receptor MRGPRX1 in sensory neurons using bimolecular fluorescence complementation (BiFC), calcium and cAMP imaging, siRNA knockdown, and pharmacological inhibition assays. Behavioral assays in mice were conducted to assess scratching responses. We also employed immunohistochemistry, RT-qPCR, and ELISA to evaluate gene and protein expression levels in dorsal root ganglia (DRG) and skin tissues, including a mouse model of atopic dermatitis (AD).

**Results:**

OPRM1 formed heterodimers with MRGPRX1 in HEK293T cells and sensory neurons, triggering a signaling switch from Gα_i/o_-mediated cAMP inhibition to Gα_q/11_-driven calcium mobilization upon activation with DAMGO or endogenous opioids. This heterodimerization elicited robust intracellular calcium responses and scratching behavior in mice, which were attenuated by OPRM1 or MRGPRX1 antagonists. In the AD mouse model, increased OPRM1 expression and β-endorphin levels were observed in DRG neurons, correlating with heightened scratching and calcium responses. In contrast, although the δ-opioid receptor (OPRD1) associated with MRGPRX2, it did not trigger mast cell degranulation, suggesting a limited contribution to peripheral itch signaling.

**Conclusions:**

Our findings identify a novel peripheral mechanism of opioid-induced itch mediated by OPRM1/MRGPRX1 heterodimers in sensory neurons. This receptor complex promotes calcium signaling and itch behavior, distinct from central or mast cell–dependent pathways. Targeting this heterodimer may offer new therapeutic strategies to alleviate opioid-induced itch without impairing analgesia.

**Supplementary Information:**

The online version contains supplementary material available at 10.1186/s12929-026-01238-x.

## Introduction

Opioids are potent analgesics widely used in the management of moderate to severe pain. Despite their effectiveness, opioid use is often complicated by substantial risks of dependence and addiction, along with a range of adverse effects. These side effects include respiratory depression, nausea, sedation, and sensory disturbances such as hyperalgesia (increased sensitivity to pain) and pruritus (itch) [1, 2]. Thus, opioid therapy requires careful management to effectively balance pain relief against potential discomfort and associated health risks.

Pruritus is an especially distressing side effect of opioid use. While pain acts as a warning signal for potential tissue damage, itch typically serves as a protective response against irritants. Opioid-induced pruritus, however, can be particularly intense and persistent, causing significant discomfort and substantially reducing the quality of life for patients. In fact, pruritus often limits the clinical utility of opioids because it can be more troubling than the pain itself [3]. Although pain and itch are mediated by similar populations of sensory neurons, the precise mechanisms underlying opioid-induced itch remain not fully understood.

The potent analgesic effects of opioids are primarily mediated by the activation of specific opioid receptors. These receptors are members of the G-protein-coupled receptor (GPCR) family and include the μ-opioid receptor (OPRM1), δ-opioid receptor (OPRD1), κ-opioid receptor (OPRK1), and opioid receptor-like 1 (ORL-1) [4]. Each receptor subtype plays a distinct role in modulating pain and other physiological responses, collectively forming a critical component of the pain management system. While activating these receptors enables opioids to exert powerful pain-relieving effects, this same mechanism is also associated with the diverse side effects, including pruritus, that are commonly observed in opioid therapy.

Numerous studies have explored the mechanisms underlying opioid-induced itch, with most focusing on the central nervous system. Notably, opioid-induced pruritus has been attributed to central OPRM1, rather than to other opioid receptor subtypes or histamine pathways [5]. Further research revealed that opioid-induced itch is mediated by the heterodimerization of a specific OPRM1 isoform with the gastrin-releasing peptide receptor (GRPR) in the spinal cord, triggering itch through the unidirectional cross-activation of GRPR signaling [6]. Additionally, intrathecal administration of the OPRM1 agonist morphine induces itch by acting on OPRM1 expressed in spinal inhibitory interneurons, resulting in the disinhibition of the central itch circuit [7]. These findings establish that central OPRM1 plays a pivotal role in mediating opioid-induced pruritus, particularly when opioids are administered neuraxially (e.g., intrathecally or epidurally).

Although central OPRM1 activation is recognized as a potential cause of opioid-induced itch, emerging evidence suggests that additional mechanisms may also contribute. Indeed, recent studies implicate peripheral opioid receptors—particularly those located in sensory nerves, mast cells, and keratinocytes near the skin—as contributing factors to opioid-induced pruritus [5, 8–10]. However, the precise mechanisms by which opioid-induced pruritus occurs at the peripheral level remain largely unknown. Therefore, a deeper understanding of peripheral opioid receptor signaling in pruritus is essential to develop strategies that manage this adverse effect while preserving the analgesic benefits of opioids.

Mas-related G protein-coupled receptors (MRGPRs), a unique GPCR family expressed on sensory neurons, have emerged as key players in modulating itch and pain signaling pathways. For instance, MRGPRX1 responds to the peptide BAM8-22, triggering itch behaviors when activated [11]. Another key receptor, MrgprA3, is activated by pruritogens such as chloroquine, and its stimulation is linked to scratching responses in animal models, highlighting its essential role in itch pathways [11, 12]. These findings underscore the role of MRGPRs in mediating peripheral itch signaling, particularly in response to histamine-independent pruritogens.

Notably, opioid receptors—particularly OPRM1—are well known to form heterodimeric complexes with various other GPCRs, resulting in altered pharmacological properties [4, 13, 14]. Such heterodimers can display modified ligand-binding affinities, distinct downstream signaling pathways, and unique functional responses compared to the individual receptor components. Of particular interest, OPRM1 has been reported to form a heterodimeric complex with MRGPRX1 (also referred to as MrgprC11 or MrgC11) [15]. This OPRM1/MRGPRX1 complex has been shown to enhance morphine-induced analgesia, suggesting a functional contribution to pain modulation [15]. However, whether this heterodimer participates in opioid-induced itch has not yet been explored.

Given emerging evidence implicating peripheral opioid receptors and their interactions with MRGPRs in opioid-induced pruritus, this study aims to elucidate the role of opioid receptor–MRGPR heterodimers in mediating peripheral itch.

## Materials and methods

### Reagents

The following reagents were purchased from Sigma-Aldrich (Seoul, Korea): [D-Ala^2^, N-Me-Phe^4^, Gly^5^-ol]-Enkephalin (DAMGO), Leucine-Enkephalin (Leu-Enk), Naltrexone, Berbamine, U73122, Pertussis toxin (PTX), MC903 (also known as calcipotriol), Compound 48/80 (C48/80), Bovine adrenal medulla 8–22 (BAM8–22), QWF, and Forskolin. β-endorphin, Endomorphin-1, Endomorphin-2, [D-Pen^2^, D-Pen^5^]-Enkephalin (DPDPE) were purchased from Tocris Bioscience (Bristol, UK). YM254890 was purchased from Wako (Osaka, Japan).

### Genes

The following cDNAs were obtained from Addgene (Watertown, MA, USA) in the study: human *OPRM1*, human *MRGPRX1*, human *OPRD1*, and human *MRGPRX2.* Mouse cDNAs in *MrgprX1* [16], *MrgprB2* [17], mouse *Oprm1*, and mouse *Oprd1* were cloned in-house, and their functions were validated. Sanger sequencing confirmed 100% identity with the corresponding sequences in the NCBI GenBank database. All cDNAs were subcloned into pcDNA3.1 expression vector for calcium imaging experiments.

### Cell culture and gene transfection

HEK293T cells were maintained in Dulbecco’s Modified Eagle Medium (DMEM; Life Technologies Corporation, NY, USA) supplemented with 10% fetal bovine serum (FBS) and 1% ZellShield® (Minerva Biolabs, Berlin, Germany). The cells were plated at a density of 3 × 10^4^ cells per well in 8-well plates pre-coated with poly-L-lysine. After 24 h, transfection was performed using FuGENE® HD Transfection Reagent (Promega, WI, USA) according to the manufacturer’s instructions. Calcium imaging was conducted 24 h post-transfection.

### Primary culture of mouse dorsal root ganglia (DRG) neurons

DRG neurons were isolated and cultured using previously described methods [18]. Briefly, the neurons were incubated at 37 °C with 1.2 mg/mL collagenase (Worthington Biochemical, Lakewood, NJ, USA) for 40 min, followed by an additional 40-min incubation at 37 °C with 2.5 mg/mL trypsin (Gibco). After incubation, the cells were centrifuged at 30×*g* for 10 min and resuspended in Gibco Neurobasal™-A Medium (Thermo Fisher Scientific, Waltham, MA, USA) supplemented with 10% FBS, 50–100 ng/mL nerve growth factor (Invitrogen, Gaithersburg, MD, USA), and 100 U/mL ZellShield®. The cells were plated at a density of 3 × 10^4^ cells per well in 8-well Lab-Tek chambers (Thermo Fisher Scientific) pre-coated with poly-L-lysine and incubated for 48 h at 37 °C under 95% humidity and 5% CO₂.

### Knockdown of *MrgprX1* in DRG neurons using small interfering RNA (siRNA)

For *MrgprX1* knockdown, DRG neurons were maintained in Gibco Neurobasal™-A Medium supplemented with 10% FBS and 1% ZellShield®. After 24 h, transfection was performed using FuGENE® SI Transfection Reagent (Promega, WI, USA) according to the manufacturer’s instructions. *MrgprX1* knockdown was achieved by custom-designed synthetic *MrgprX1*-specific siRNA (Bioneer, Daejeon, Korea). The sequences are 5′-CAG-GAA-ACA-CCU-UUG-UAC-dTdT-3′ (sense), and 5′-AGU-ACA-AUG-GUG-UUU-CCU-G-dTdT-3′ (anti-sense). For negative control, AccuTarget™ NC siRNA (#SN-1022, Bioneer) was used. Calcium imaging was conducted 24 h post-transfection to evaluate knockdown efficiency and functional responses.

### Calcium imaging

Intracellular calcium levels were measured using a calcium-specific fluorescent dye with a fluorescence microscope (DMi8, Leica, Wetzlar, Germany). Briefly, cells were loaded with 5 μM Fluo-3/AM (Invitrogen, Eugene, USA) and incubated at 37 °C for 40 min. Subsequently, the cells were washed with 1 × NBS solution (140 mM NaCl, 5 mM KCl, 2 mM CaCl_2_, 0.5 mM MgCl_2_, 10 mM glucose, 5.5 mM HEPES, adjusted to pH 7.4). The excitation wavelength was 488 nm, and the emission fluorescence was recorded at 515 nm. The microscopic images were captured at an interval of 1.5 s, using a connected computer. The changes in fluorescence intensity, which reflect the intracellular calcium levels, were expressed as an F/F_0_ ratio, where F is the fluorescence intensity at a given time and F_0_ is the fluorescence intensity at time zero. The images were analyzed using ImageJ (NIH) with custom-made scripts for automatic cell count and pseudoratiometric image production. The number of* n* in the calcium imaging results indicate the total cell count collected from at least three independent calcium imaging experiments.

### Cyclic AMP (cAMP) imaging

Intracellular cAMP levels were monitored using G-Flamp1 [19], a genetically encoded fluorescent indicator specifically designed for cAMP detection. The procedures were largely identical to those used for calcium imaging, with the exception that the G-Flamp1 (plasmid #188567, Addgene) was co-transfected along with the target gene constructs. As G-Flamp1 utilizes cAMP-sensitive fluorescent proteins, the loading step with Fluo-3/AM was omitted. Fluorescence signal measurements were conducted using the same microscopy and analysis configuration as described in the calcium imaging method.

### Biomolecular fluorescence complementation (BiFC) assay

The BiFC assay was conducted to investigate potential interactions between OPRM1 and MRGPRX1. For this purpose, VN and VC, two complementary fragments of the fluorescent protein Venus, were utilized to monitor interactions between these proteins. Specifically, the coding sequences of human *OPRM1* and mouse *Oprm1* were fused into pBiFC-VN173 (plasmid #22010, Addgene), while those of human *MRGPRX1* and mouse *MrgprX1* were fused into pBiFC-VC155 (plasmid #22011, Addgene). Sanger sequencing verified that the fused constructs were 100% identical to the reference sequences. The constructs were transfected into HEK293T cells, and Venus fluorescence was detected using the same microscope and configuration described in the calcium imaging section.

### Mouse scratching behavior test

All animal experiments were conducted in accordance with the approved animal protocol (GIACUC-R202110-01, GU1-2023-IA0026) by the Institutional Animal Care and Use Committee of Gachon University, Incheon, Korea. Six C57BL/6 mice, aged between 9 and 14 weeks, were randomly assigned to either the control or treatment group. The mice were maintained under a 12:12 h light:dark cycle and provided ad libitum access to food and water. The scratching behavior of mice was video recorded for up to 1 h after the injection, and the number and duration of scratching bouts were counted using the Solomon Coder program (https://solomon.andraspeter.com). One bout of scratching was defined as the movement of hind limbs near the cheek until the mouse touches the floor.

### Generation of an MC903-induced AD-like animal model

To induce an atopic dermatitis (AD)–like skin condition, C57BL/6 mice (9–14 weeks old; Koatech, Pyeongtaek, Korea) were used. The dorsal nape region was shaved three days before treatment. MC903 ointment (Daivonex; LEO Pharma, Ballerup, Denmark; 40 mg per mouse) was topically applied once daily to the shaved area for 14 consecutive days. This regimen reliably produced key AD-like features, including erythema, desquamation, and thick white scaly plaques.

### Immunohistochemistry

DRG sections were cut using a cryostat (10 μm thick) and attached to the slides. The tissue sections were washed with phosphate-buffered saline (PBS) and fixed with 4% paraformaldehyde for 10 min. Hydrogen peroxide (1%) was used to suppress endogenous peroxidase activity, and 0.3% Triton X-100 with 1% FBS was used for blocking. The samples were then incubated overnight at 4 °C with MRGPRX1 rabbit polyclonal antibody (Invitrogen, PA1-20496) at 1:80 dilution and µ-opioid receptor guinea pig polyclonal antibody (Neuromics, GP10106) at 1:80 dilution. The next day, the samples were washed with PBS and incubated with goat anti-rabbit IgG H&L secondary antibody (Alexa Fluor® 488; Abcam, ab150077) at 1:200 dilution and goat anti-guinea pig IgG H&L secondary antibody (Alexa Fluor® 647; Abcam, ab150187) at 1:200 dilution for 2 h in the dark to avoid photobleaching. Primary and secondary antibodies were prepared in 0.3% Triton-X 100 containing 0.5% FBS. After washing the samples with PBS, freshly prepared DAPI staining solution in PBS was added, and the samples were incubated for 10 min in the dark. The samples were rinsed two to three times with PBS, mounted with VECTASHIELD® (Vector Laboratories, Burlingame, CA, USA) and covered with a coverslip. The slides were visualized, and images were obtained using a Leica DMi8 inverted microscope (Leica Microsystem Ltd., Wetzlar, Germany).

### Enzyme-linked immunosorbent assay (ELISA)

The levels of β-endorphin were measured using a mouse β-endorphin ELISA kit (catalog # CSB-E06827m; CUSABIO, Houston, USA), following the manufacturer’s protocols.

### Real-time quantitative polymerase chain reaction (RT-qPCR)

Total RNA was extracted using Total RNA Extraction Kit (iNtRON, Gyeonggi-do, Korea). First‐strand cDNA was synthesized with PrimerScript™ RT Master Mix (TaKaRa, Shiga, Japan). RT-qPCR was then performed on QuantStudio 1 qPCR System (ThermoFisher, Gangnam, Korea) using TB Green® Premix Ex Tag™ II (Tli RNaseH Plus) and ROX Plus (#RR82LR; TaKaRa, Shiga, Japan). The cycling conditions were as follows: 95 °C for 30 s (denaturation), 55 °C for 60 s (annealing), and 72 °C for 60 s (extension with fluorescence detection), repeated for 40 cycles. Glyceraldehyde 3-phosphate dehydrogenase (*Gapdh*) served as the internal control. The primer sequences used for the reactions are as follows: *Gapdh* (forward: 5′-AGG-TCG-GTG-TGA-ACG-GAT-TT-3′, reverse: 5′-TGT-AGA-CCA-TGT-AGT-TGA-GG-3′), *Oprm1* (forward: 5′-TCC-GAC-TCA-TGT-TGA-AAA-ACC-C-3′, reverse: 5′-CCT-TCC-CCG-GAT-TCC-TGT-CT-3′), *Mrgprx1* (forward: 5′-TCT-CAT-CCC-ACG-ACA-CAG-AAT-3′, reverse: 5′-AGC-CAG-AGT-ACA-ATG-GTG-TTT-C-3′), *Trpv1* (forward: 5′-CCA-CTG-GTG-TTG-AGA-CGC-C-3′, reverse: 5′-TCT-GGG-TCT-TTG-AAC-TCG-CTG-3′), and *Trpa1* (forward: 5′-GTC-CAG-GGC-GTT-GTC-TAT-CG-3′, reverse: 5′-AGC-ACT-TCA-CAC-GAA-GAA-CCA-3′).

### HMC-1.2 human mast cell line culture

HMC-1.2 cells (#SCC062; Millipore, Burlington, Massachusetts, USA) were cultured in Roswell Park Memorial Institute (RPMI) 1640 medium (Gibco, ThermoFisher, Gangnam, Korea), supplemented with 10% heat-inactivated FBS, 1.2 mM α-thioglycerol (Sigma-Aldrich), and 1% ZellShield® to prevent contamination. The cells were plated in 25T flasks at a density of 4 × 10^6^ cells per well and maintained under standard culture conditions before proceeding with the β-hexosaminidase assay.

### Primary culture of peritoneal mast cell (PMC)

Primary culture of PMCs was performed as previously described [18]. Briefly, the mice were euthanized with CO_2_ prior to experimentation. The skin of the abdomen was incised, and 7 mL of ice-cold RPMI 1640 medium was added for detaching the PMCs from the peritoneum. The media containing the PMCs was centrifuged at 300×*g* for 5 min, and the PMCs were seeded in 25 T flasks with murine IL-3 (mIL-3, 10 ng/mL; Sigma-Aldrich) and murine stem cell factor (mSCF, 30 ng/mL; Peprotech, NJ, USA). After 48 h of incubation, the medium was replaced, and mIL-3 and mSCF were added again. The PMCs were cultured for 12 days until experimentation.

### β-hexosaminidase assay

HMC-1.2 cells or PMCs were transferred to 96-well V-bottom plates and incubated at 37 °C in a 5% CO_2_ incubator for 1 h. Following the initial incubation, C48/80 (100 µg/mL), DPDPE (10–100 µM), and Leu-Enk (10–100 µM) were added to the wells. The cells were incubated for an additional 1 h under the same conditions. After incubation, the plates were centrifuged at 120×*g* for 5 min at 4 °C. From each well, 195 μL of the supernatant was carefully collected and transferred to a clean 96-well flat-bottom plate, which was kept on ice until further analysis. To the remaining cell pellets in the V-bottom plate, 200 μL of lysis buffer (1% Triton X-100 in 1 × NBS) was added, followed by incubation at room temperature for 5 min. The pellets were then resuspended by gentle pipetting, and the lysates were prepared. In a new flat-bottom 96-well plate, 25 μL of 4 mM p-nitrophenyl-N-acetyl-β-D-glucosaminide (pNAG; dissolved in 0.4 M citric acid, pH 4.5) was added to each well, along with 25 μL of either the supernatant or lysate. The plate was incubated at 37 °C for 1 h. After incubation, 150 μL of stop solution (200 mM glycine, pH adjusted to 10.7) was added to each well to terminate the reaction. The absorbance of the supernatant and lysate was measured at 405 nm using a microplate reader (Synergy H1 Hybrid Reader, BioTek, VT, USA). The percentage of β-hexosaminidase release, an indicator of mast cell degranulation, was calculated using the following formula:

$${\mathrm{Release}}\left(\mathrm{\%}\right) \mathrm{=} \frac{{\mathrm{A}}_{\mathrm{supernatant}}}{{\mathrm{A}}_{\mathrm{supernatant}}\text{ } + {\text{ A}}_{\mathrm{lysate}}}\times{100}$$

where Release (%) represents the percentage of β-hexosaminidase released into the supernatant relative to the total β-hexosaminidase content.

### Statistical analysis

All statistical analyses were performed using GraphPad Prism software (version 9). Error bars in the bar graph represent the mean ± standard error, whereas those in the time-course calcium imaging graphs indicate 95% confidence intervals. Unpaired t-tests were used for comparison between two groups and one-way analysis of variance (ANOVA) with Dunnett’s multiple comparison post-test was used for groups of more than three. Fisher's exact test was used to compare the responsiveness of the primarily cultured DRG neurons. A *p* value of less than 0.05 was considered statistically significant.

## Results

### OPRM1 forms a heterodimer with MRGPRX1

**Fig. 1:**
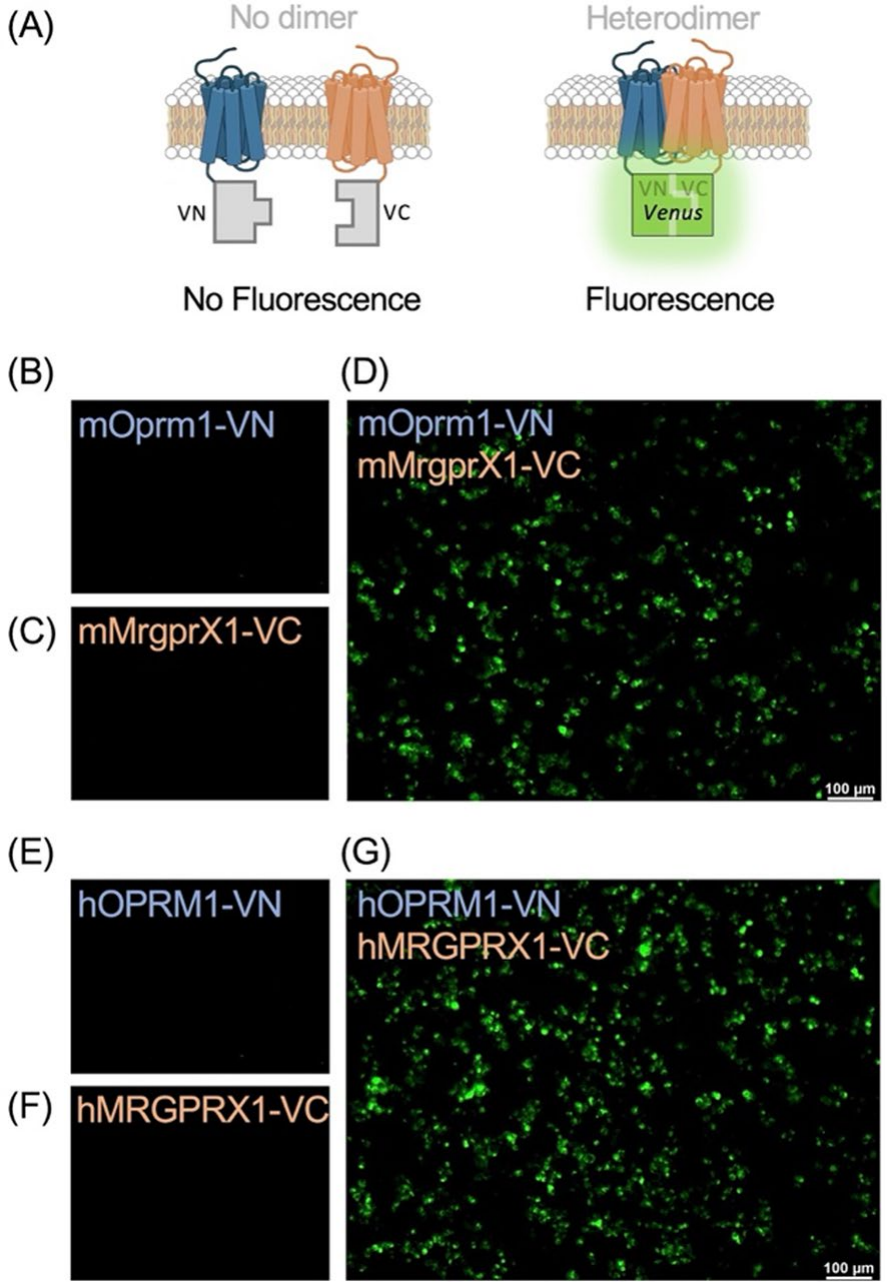
Biomolecular fluorescence complementation (BiFC) analysis of OPRM1 and MRGPRX1 heterodimer formation. **A** Schematic of the BiFC assay used to test heterodimer formation. Venus fragments (VN and VC) were fused to OPRM1 and MRGPRX1, respectively. Fluorescence appears only upon successful heterodimerization. **B**–**D** Microscopic images of HEK293T cells expressing mouse Oprm1-VN alone (**B**), mouse MrgprX1-VC alone (**C**), or both constructs together (**D**). Fluorescence is observed only in co-transfected cells. **E**–**G** Microscopic images of HEK293T cells expressing human OPRM1-VN alone (**E**) or human MRGPRX1-VC alone (**F**) show no fluorescence. Fluorescence is detected only upon co-expression (**G**). Scale bar = 100 μm

To confirm potential heterodimer formation between OPRM1 and MRGPRX1, we employed a bimolecular fluorescence complementation (BiFC) assay in HEK293T cells. In this assay, two non-fluorescent *Venus* fragments (VN and VC) generates fluorescence only when brought into close proximity by interacting fusion partners [20] (Fig. 1A). Mouse *Oprm1* and *MrgprX1* were N-terminally fused to VN and VC, respectively (mOprm1-VN and mMrgprX1-VC). When expressed individually, neither mOprm1-VN (Fig. 1B) nor mMrgprX1-VC (Fig. 1C) exhibited fluorescence, confirming the absence of spontaneous interaction or self-assembly. However, robust fluorescence was observed upon co-expression of mOprm1-VN and mMrgprX1-VC in HEK293T cells (Fig. 1D), indicating heterodimeric complex formation.

Similarly, human ortholog fusion constructs, *OPRM1* with VN (hOPRM1-VN) and *MRGPRX1* with VC (hMRGPRX1-VC), showed no fluorescence when expressed individually (Fig. 1E and F) but co-expression yielded robust fluorescence (Fig. 1G). Thus, these findings demonstrate that OPRM1 and MRGPRX1 can form heterodimeric complexes in HEK293T cells.

### Functional expression and signaling characterization of OPRM1 and MRGPRX1

OPRM1 is a GPCR that primarily signals through the G protein alpha subunit Gα_i/o_, which leads to inhibition of adenylyl cyclase activity and a consequent decrease in intracellular cyclic AMP (cAMP) levels upon agonist stimulation. The functionality of OPRM1 was validated using G-Flamp1, a genetically encoded fluorescent biosensor whose emission intensity correlates with intracellular cAMP levels [21]. An increase in cAMP should result in higher G-Flamp1 fluorescence (Fig. S1A). Indeed, cells expressing G-Flamp1 showed a significant increase in fluorescence upon treatment with forskolin, an adenylyl cyclase activator known to elevate intracellular cAMP (Fig. S1B). The effects of DAMGO, an OPRM1 agonist, on cAMP levels were then examined in cells transfected with either mOprm1 or mMrgprX1. Treatment with 10 μM DAMGO led to a gradual decrease in fluorescence in cells transfected with mOprm1 (Fig. S1C), implying that activation of OPRM1 by DAMGO successfully reduced intracellular cAMP levels through the canonical Gα_i/o_ signaling pathway. However, DAMGO had no effect on fluorescence in cells transfected with mMrgprX1 (Fig. S1C), confirming that MRGPRX1 does not modulate cAMP production.

Meanwhile, it is reported that MRGPRX1 is preferably coupled to Gα_q/11_ [22], which stimulates phospholipase C (PLC) to hydrolyze the membrane lipid phosphatidylinositol 4,5-bisphosphate (PIP_2_) into inositol 1,4,5-trisphosphate (IP_3_) and diacylglycerol (DAG). IP_3_ subsequently triggers the release of calcium from intracellular stores, leading to increased cytosolic calcium levels. To confirm functional expression and Gα_q/11_ coupling of MRGPRX1, calcium imaging experiments were conducted using the fluorescent calcium indicator dye Fluo-3/AM (Fig. S1D). As expected, treatment with BAM8-22, an MRGPRX1 agonist, produced a significant increase in fluorescence in cells transfected with mMrgprX1 (Fig. S1E), indicating elevated intracellular calcium levels mediated by the canonical Gα_q/11_ signaling cascade upon MRGPRX1 activation. As expected, BAM8-22 did not alter calcium levels in cells transfected with mOprm1 (Fig. S1E).

Overall, these results confirm that OPRM1 activation by DAMGO decreases cAMP levels, while MRGPRX1 stimulation by BAM8-22 increases intracellular calcium, validating the functional expression and canonical signaling mechanisms of both receptors.

### Crosstalk between OPRM1 and MRGPRX1 mediates opioid-induced calcium mobilization

**Fig. 2:**
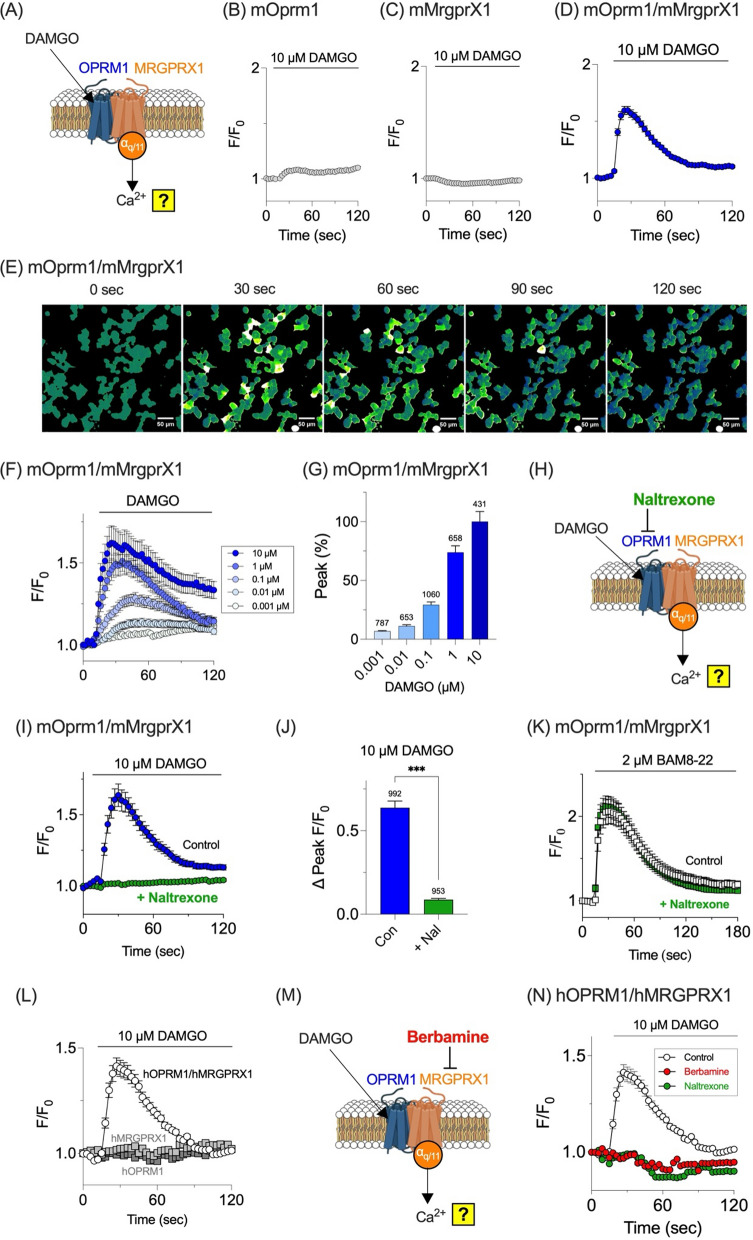
OPRM1 and MRGPRX1 heterodimer induces DAMGO-induced calcium mobilization. **A** Schematic representation of a calcium imaging assay used to verify OPRM1/MRGPRX1 heterodimer function and determine whether DAMGO binding to OPRM1 within the heterodimer triggers an increase in intracellular calcium via the MRGPRX1-linked Gα_q/11_ pathway. **B**–**D** Intracellular calcium changes (F/F₀) in HEK293T cells expressing **B** mOprm1 (n = 1321), **C** mMrgprX1 (n = 738), or **D** both mOprm1 and mMrgprX1 (mOprm1/mMrgprX1; n = 3294) upon treatment with 10 µM DAMGO. Note that calcium increase is observed only in **D**. **E** Representative pseudocolor images of calcium responses in mOprm1/mMrgprX1 cells at various time points (0–120 s) following DAMGO stimulation. Brighter colors indicate elevated calcium concentrations. Scale bar = 50 µm. **F** Dose–response graphs of DAMGO-induced calcium changes in mOprm1/mMrgprX1 cells. Peak calcium mobilization increases with higher DAMGO concentrations. **G** Quantification of peak responses (percentage of maximum signal) at various DAMGO concentrations. The numbers displayed above the graph represent the sample sizes (n). **H** Inhibition assay schematic. Pretreatment with naltrexone will block DAMGO engagement of OPRM1 in the heterodimer, which may prevent downstream Gα_q/11_–mediated calcium mobilization. **I** DAMGO-induced calcium responses in mOprm1/mMrgprX1 cells are significantly inhibited by naltrexone. **J** Quantified peak changes (ΔPeak F/F₀) of (I) in the absence (Con) and presence of naltrexone (+ Nal). The numbers displayed above the graph represent the sample sizes (n). **K** BAM8-22-induced calcium responses in mOprm1/mMrgprX1 cells (n = 560) are unaffected by an OPRM1 inhibitor naltrexone (n = 731). **L** DAMGO-induced calcium mobilization was only observed in hOPRM1/hMRGPRX1 cells (n = 2256), but not in cells expressing hOPRM1 (n = 2207) or hMRGPRX1 (n = 1244) alone. **M** Inhibition assay schematic. Pretreatment with berbamine antagonizes MRGPRX1 within the heterodimer, which may abrogate DAMGO-induced Gα_q/11_–dependent calcium increase. **N** DAMGO-induced calcium responses in hOPRM1/hMRGPRX1 cells (n = 2256) are inhibited by berbamine (an MRGPRX1 antagonist; n = 1731) or naltrexone (an OPRM1 antagonist; n = 1681). ***p < 0.001

Next, whether OPRM1/MRGPRX1 heterodimerization alters signaling pathways was examined. Specifically, the effect of OPRM1 activation by DAMGO on MRGPRX1-mediated intracellular calcium changes was assessed (Fig. 2A). Treatment of cells expressing mOprm1 alone with 10 µM DAMGO did not induce an increase in intracellular calcium levels (Fig. 2B). This is expected because activation of the Gα_i/o_-coupled OPRM1 by DAMGO reduces cAMP levels rather than modulating intracellular calcium signaling. Similarly, DAMGO treatment of mMrgprX1-expressing cells did not alter intracellular calcium levels, as DAMGO does not activate the Gα_q/11_-coupled MRGPRX1 (Fig. 2C). Surprisingly, when mOprm1 and mMrpgrX1 were co-transfected in HEK293T cells (“mOprm1/mMrgprX1” cells), DAMGO induced a significant increase in intracellular calcium levels (Fig. 2D and E). Because DAMGO selectively activates the Gα_i/o_-coupled OPRM1 and not the Gα_q/11_-coupled MRGPRX1, the observed calcium increase indicates functional crosstalk between the two receptors. Moreover, the magnitude of the calcium response scaled with DAMGO concentration, demonstrating a dose-dependent effect (Fig. 2F and G).

To confirm OPRM1 involvement, mOprm1/mMrgprX1 cells were pretreated with the OPRM1 antagonist naltrexone prior to DAMGO exposure (Fig. 2H). Naltrexone pretreatment completely abolished the DAMGO-induced calcium increase (Fig. 2I and 2J), confirming that the response is mediated by OPRM1. In contrast, naltrexone did not inhibit MRGPRX1 activation by BAM8-22 in mOprm1/mMrgprX1 cells (Fig. 2K), demonstrating that the effect of naltrexone is specific to OPRM1.

Experiments were extended to the human orthologs. Application of 10 µM DAMGO evoked a robust increase in intracellular calcium levels only in HEK293T cells co-transfected with hOPRM1 and hMRGPRX1 (“hOPRM1/hMRGPRX1” cells), but not in cells expressing each receptor individually (Fig. 2L). To assess the role of MRGPRX1, cells were pretreated with berbamine—an antagonist of human MRGPRX1 [23]—prior to DAMGO exposure (Fig. 2M). Indeed, berbamine robustly suppressed the DAMGO-induced calcium response in hOPRM1/hMRGPRX1 cells, and naltrexone was equally effective (Fig. 2N). These results demonstrate that DAMGO-induced calcium mobilization in HEK293T cells requires co-expression of both human OPRM1 and MRGPRX1.

Overall, these findings suggest that the co-expression of OPRM1 and MRGPRX1 enables novel signaling crosstalk, whereby OPRM1 activation by DAMGO triggers calcium mobilization via MRGPRX1.

### Gα_q/11_ and phospholipase C mediate DAMGO-induced calcium mobilization in OPRM1/MRGPRX1 complex

**Fig. 3:**
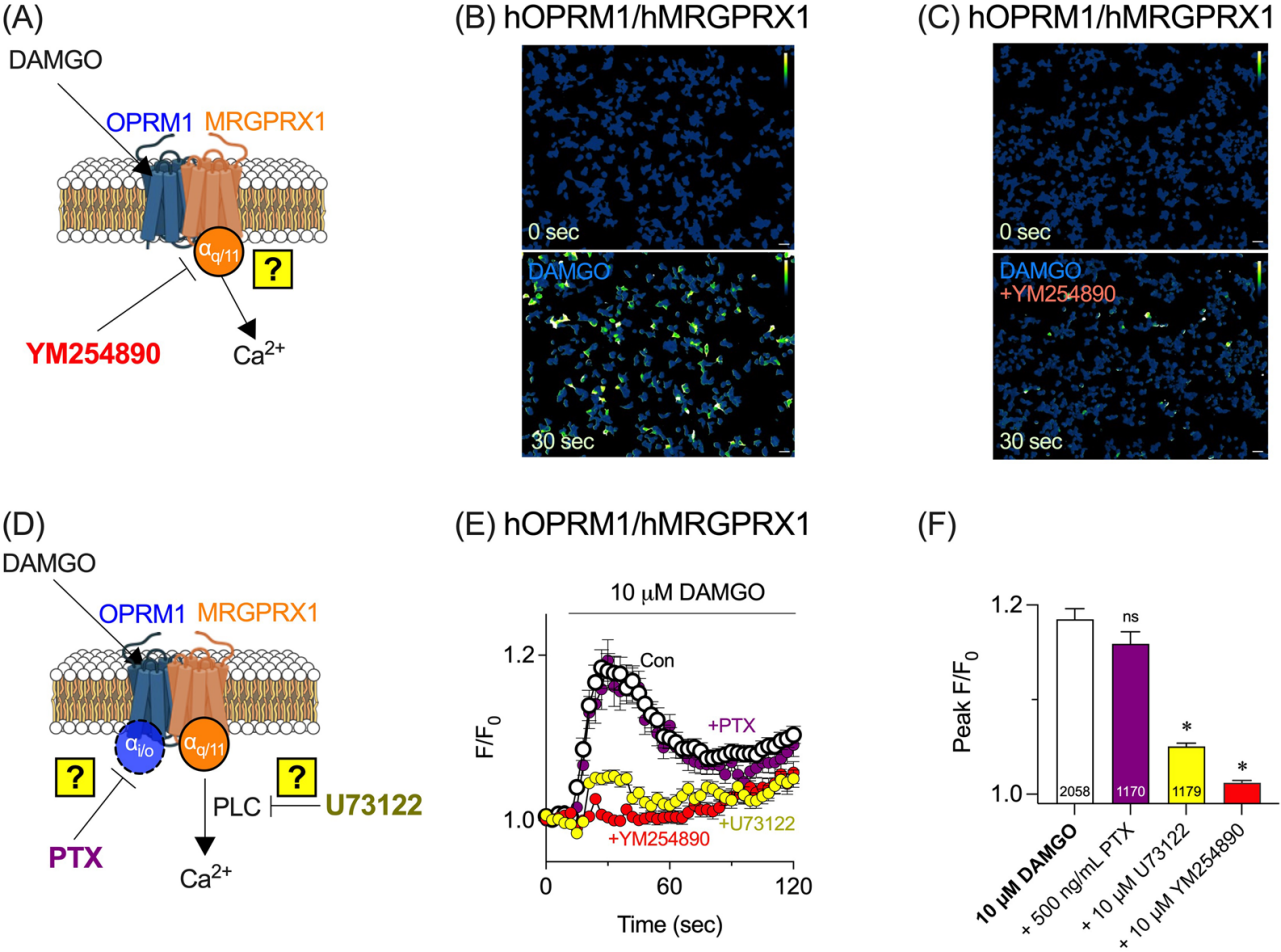
DAMGO-induced calcium mobilization in OPRM1/MRGPRX1 is mediated by Gα_q/11_ and phospholipase C. **A** Schematic of inhibition assay. YM254890, a Gα_q/11_ inhibitor, is applied prior to DAMGO to assess Gα_q/11_ involvement. **B** Representative pseudocolor images of HEK293T cells co-expressing hOPRM1 and hMRGPRX1 (hOPRM1/hMRGPRX1) before (top) and after (bottom) treatment with 10 µM DAMGO. **C** Pretreatment with 10 µM YM254890, a selective Gα_q/11_ inhibitor, significantly reduced DAMGO-induced calcium mobilization. Scale bars indicate 50 µm. **D** Schematic of inhibitor assay for Gα_i/o_ and PLC contributions. Pertussis toxin (PTX) blocks Gα_i/o_, whereas U73122 inhibits PLC. **E** Time-course of calcium responses (F/F_0_) in hOPRM1/hMRGPRX1 cells treated with DAMGO under various conditions: control, PTX (500 ng/mL, Gα_i/o_ inhibitor), U73122 (10 µM, PLC inhibitor), and YM254890 (10 µM, Gα_q/11_ inhibitor). **F** Quantification of peak responses (ΔPeak F/F₀) for the conditions in (**D**). Numbers shown inside the bars indicate the sample sizes (n). Statistical significance is indicated as follows: ns: not significant, *p < 0.05

To further confirm Gα_q/11_ involvement in the DAMGO-induced calcium increase, hOPRM1/hMRGPRX1 cells were pretreated with YM254890—a specific Gα_q/11_ inhibitor—prior to DAMGO application (Fig. 3A). As a control, 10 µM DAMGO induced a clear increase in calcium levels in hOPRM1/hMRGPRX1 cells (Fig. 3B). However, after pretreatment with 10 µM YM254890, the DAMGO-induced calcium response was strongly suppressed (Fig. 3C), confirming Gα_q/11_ mediation.

Given that OPRM1 is coupled to Gα_i/o_, its contribution to the DAMGO-induced calcium increase was evaluated (Fig. 3D). However, pretreatment with pertussis toxin (PTX), a Gα_i/o_ inhibitor, did not suppress the calcium response (Fig. 3E and F), indicating no Gα_i/o_ involvement. Furthermore, pretreatment with a phospholipase C (PLC) inhibitor U73122 (Fig. 3D) abolished the DAMGO-induced calcium rise (Fig. 3E and F), demonstrating PLC participation.

Together, these findings show that in HEK293T cells co-expressing OPRM1 and MRGPRX1, DAMGO-triggered calcium mobilization is mediated by Gα_q/11_ and PLC rather than by Gα_i/o_.

### Endogenous opioids can also activate OPRM1/MRGPRX1 complex

**Fig. 4:**
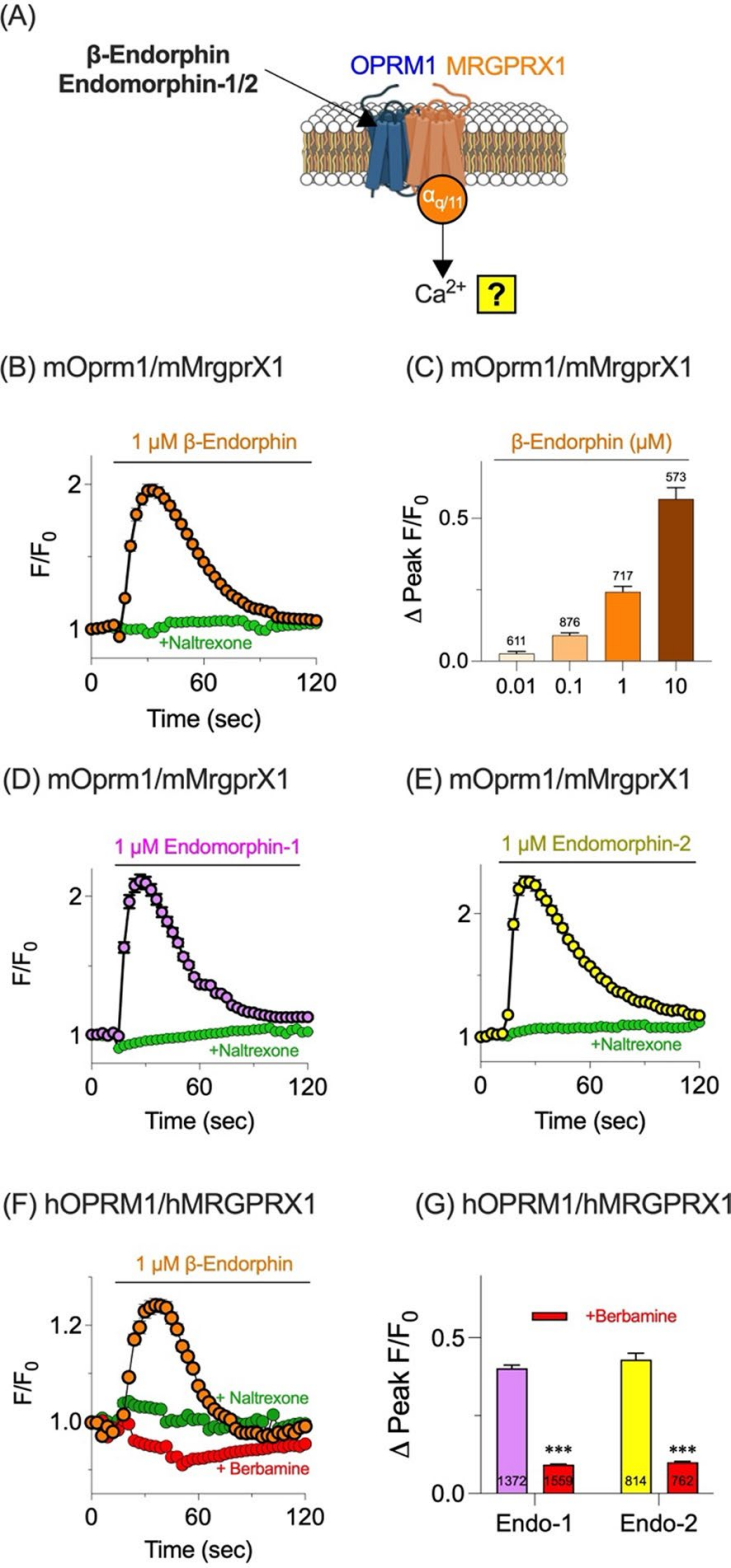
Endogenous µ-opioid agonists induce OPRM1/MRGPRX1-dependent calcium mobilization. **A** Schematic representation of a calcium imaging assay to verify whether endogenous opioids can induce intracellular calcium mobilization via OPRM1/MRGRPX1. **B** Time course of normalized fluorescence (F/F₀) in HEK293T cells co-expressing mouse Oprm1 and MrgprX1 (mOprm1/mMrgprX1) after treatment with 1 µM β-endorphin, in the absence or presence of 10 µM naltrexone. **C** Dose–response relationship showing ΔPeak F/F₀ in mOprm1/mMrgprX1 cells treated with increasing concentrations of β-endorphin (0.01–10 µM). Time-course calcium mobilization in mOprm1/mMrgprX1 cells treated with 1 µM endomorphin-1 (**D**) or endomorphin-2 (**E**), with or without naltrexone. **F** Calcium responses (F/F_0_) in HEK293T cells co-expressing hOPRM1 and hMRGPRX1 (hOPRM1/hMRGPRX1) treated with 1 µM β-endorphin in the presence of naltrexone (10 µM) or berbamine (10 µM, an MRGPRX1 antagonist). **G** Quantification of peak responses (ΔPeak F/F_0_) in hOPRM1/hMRGPRX1 cells treated with endomorphin-1 or endomorphin-2, with or without berbamine. ***p < 0.001 compared to control

Although DAMGO induces calcium influx via the OPRM1/MRGPRX1 complex, the ability of endogenous OPRM1 agonists—β-endorphin, endomorphin-1, and endomorphin-2—to elicit similar responses was assessed (Fig. 4A). In mOprm1/mMrgprX1 cells, 1 µM β-endorphin caused a significant increase in intracellular calcium, and pretreatment with naltrexone abolished this response (Fig. 4B). The β-endorphin-induced calcium rise exhibited a concentration-dependent pattern (Fig. 4C). Likewise, endomorphin-1 (Fig. 4D) and endomorphin-2 (Fig. 4E) triggered comparable calcium responses in mOprm1/mMrgprX1 cells, both of which were inhibited by naltrexone.

In hOPRM1/hMRGPRX1 cells, β-endorphin also provoked a marked calcium increase (Fig. 4F). Both naltrexone and berbamine suppressed the β-endorphin-induced response, indicating contributions from both OPRM1 and MRGPRX1. Endomorphin-1 and endomorphin-2 produced similar calcium elevations in hOPRM1/hMRGPRX1 cells, each of which was attenuated by berbamine (Fig. 4G).

These results demonstrate that endogenous µ-opioid agonists—β-endorphin, endomorphin-1, and endomorphin-2—activate the OPRM1/MRGPRX1 heterodimeric complex, supporting the concept that heterodimer activation leads to intracellular calcium mobilization.

### OPRM1/MRGPRX1 heterodimers mediate opioid-induced calcium signaling in sensory neurons

**Fig. 5:**
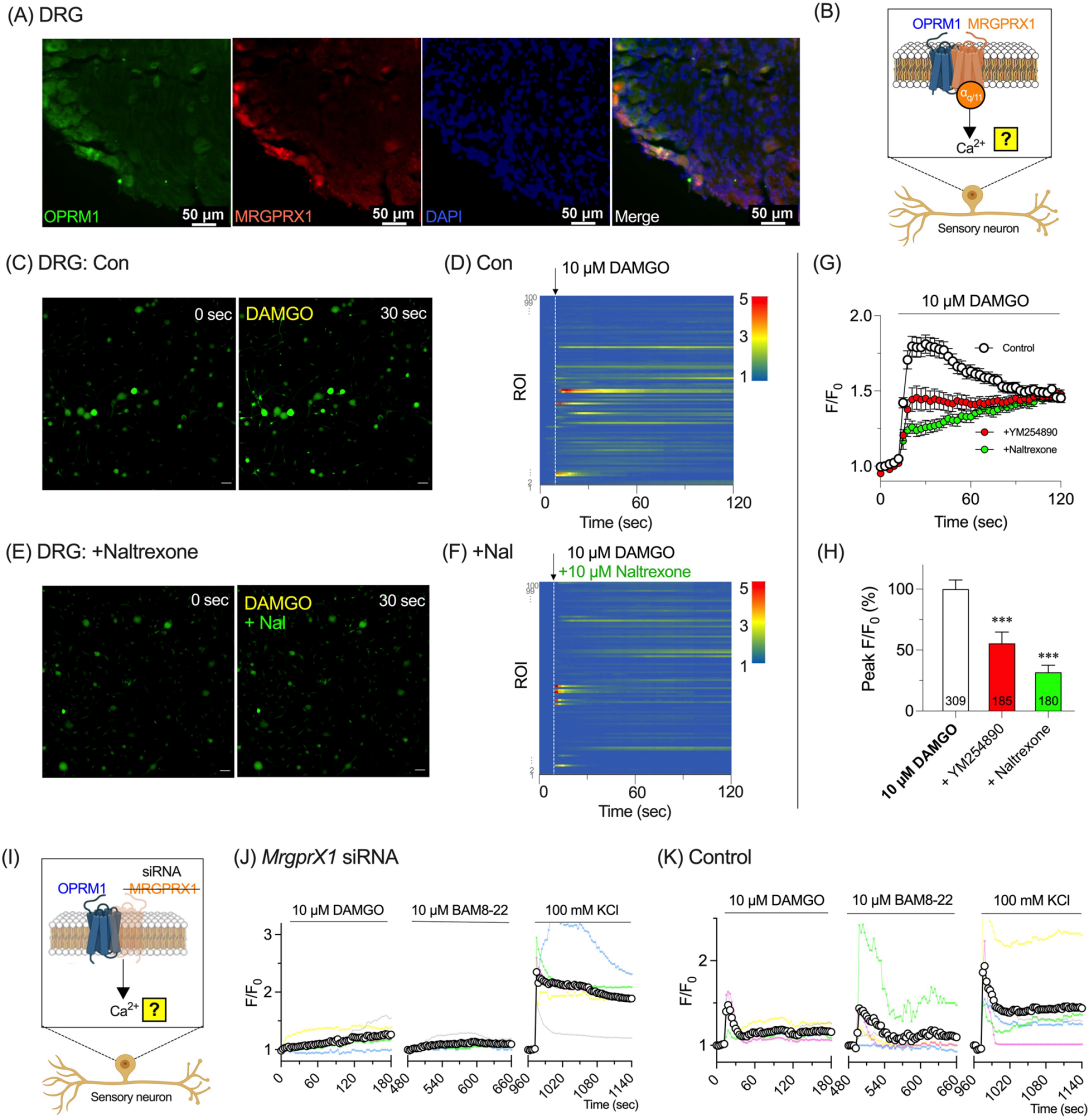
OPRM1 and MRGPRX1 induce DAMGO-induced calcium increase in mouse dorsal root ganglia (DRG) neurons. **A** Immunostaining for OPRM1 (green) and MRGPRX1 (red), with nuclear staining by DAPI (blue) in mouse DRG. The merged image demonstrates co-localization of OPRM1 and MRGPRX1 in a subset of DRG. Scale bar = 50 µm. **B** Schematic of a calcium imaging assay to verify functional presence of OPRM1/MRGPRX1 heterodimers in sensory neurons. **C** Representative images showing DAMGO-induced calcium mobilization in DRG cells under control conditions (**C**) and in the presence of naltrexone (**E**). Scale bars = 50 µm. Heatmaps showing calcium responses (F/F₀) over time across regions of interest (ROI) in DRG neurons treated with 10 µM DAMGO under control conditions (**D**) or in the presence of 10 µM naltrexone (**F**). **G** Time-course of calcium mobilization (F/F₀) in DRG cells induced by 10 µM DAMGO under various pretreated conditions: control, YM254890 (Gα_q/11_ inhibitor), and naltrexone. **H** Quantification of peak calcium responses (ΔPeak F/F₀) for the conditions in **G**. Numbers shown inside the bars indicate the sample sizes (n). ***p < 0.001 compared to control. **I** Schematic of a calcium imaging assay in sensory neurons using siRNA to verify the role of MRGPRX1 within OPRM1/MRGPRX1 heterodimers. **J**, **K** Calcium responses (F/F₀) in DRG cells treated with 10 µM DAMGO, 10 µM BAM8-22, or 100 mM KCl under *MrgprX1* knockdown conditions (**J**) and control conditions (**K**)

Because itch is transmitted by peripheral afferent neurons, receptor co-expression in mouse dorsal root ganglion (DRG) neurons was analyzed to evaluate potential OPRM1/MRGPRX1 heterodimer formation. In silico analysis of unbiased RNA-sequencing datasets [24] revealed that *Oprm1* is predominantly expressed in NP2, NP3, PEP1, and PEP2 dorsal root ganglion (DRG) neuron populations (NP refers to non-peptidergic, whereas PEP denotes peptidergic), whereas *MrgprX1* expression is restricted to NP2 and NP3 subtypes (Fig. S2). These overlapping expression patterns suggest possible concurrent expression of OPRM1 and MRGPRX1 in NP2 and NP3 neurons—subsets known to mediate pruritus [24]. Indeed, co-expression of OPRM1 and MRGPRX1 in DRG neurons was confirmed by immunohistochemistry (Fig. 5A), which identified a subset of neurons positive for both receptors.

The functional activity of the heterodimer in primary mouse DRG cultures was then assessed (Fig. 5B). DAMGO application to DRG cultures triggered an increase in intracellular calcium (Fig. 5C and 5D), an effect that was abolished by naltrexone (Fig. 5E and F) and attenuated by the Gα_q/11_ inhibitor YM254890 (Fig. 5G and H), indicating involvement of OPRM1 and downstream Gα_q/11_–PLC signaling.

To confirm that MRGPRX1 is essential for these calcium changes, gene-specific knockdown of *MrgprX1* was performed in DRG neurons using siRNA, and subsequent calcium imaging experiments were conducted (Fig. 5I). The results showed that *MrgprX1* knockdown significantly reduced DAMGO-induced calcium rises (Fig. 5J) compared with control groups (Fig. 5K), confirming MRGPRX1 involvement. These findings support a model in which an OPRM1/MRGPRX1 heterodimer mediates opioid-induced calcium signaling in peripheral sensory neurons.

### Peripheral OPRM1 upregulation and elevated β-endorphin drive enhanced opioid-induced itch in an AD model

**Fig. 6:**
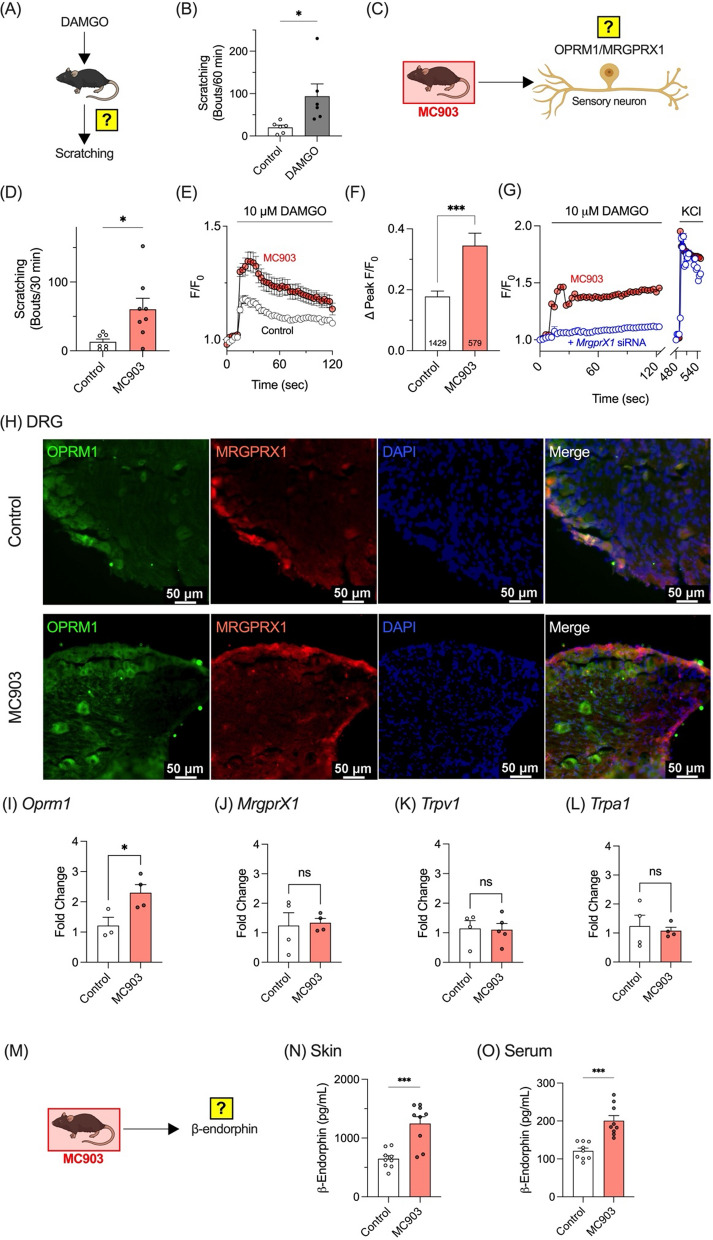
Peripheral OPRM1 mediates opioid-induced and atopic dermatitis–associated itch. **A** Schematic of intradermal DAMGO injection and subsequent scratching behavior assay. **B** Quantification of scratching bouts over 60 min in wild-type mice following intradermal injection of vehicle (Control, n = 6) or DAMGO (n = 7). **C** Schematic of investigation into the effect of MC903 treatment on OPRM1/MRGPRX1 heterodimers in sensory neurons. **D** Spontaneous scratching bouts measured over 30 min in control (n = 6) and MC903-treated mice (n = 8). **E** Time-course calcium mobilization (F/F₀) in dorsal root ganglion (DRG) neurons from wild-type (WT) and MC903-treated mice stimulated with 10 µM DAMGO. **F** Quantification of peak calcium responses (ΔPeak F/F₀) in DRG neurons from control and MC903-treated mice stimulated with DAMGO. Numbers shown inside the bars indicate the sample sizes (n). **G** DAMGO-evoked calcium signals in MC903 DRG neurons (n = 787) were significantly diminished by *MrgprX1* siRNA (n = 580). KCl (100 mM) was applied to confirm neuronal viability. **H** Immunofluorescence micrographs of lumbar DRG sections from control (identical to Fig. 5A) and MC903-treated mice. OPRM1 (green), MRGPRX1 (red), and nuclei (DAPI, blue) merge to reveal colocalization. Scale bar = 50 µm. **I**–**L** Quantification of gene expression changes (fold change) in DRG neurons from MC903-treated mice for *Oprm1* (**I**), *Mrgprx1* (**J**), *Trpv1* (**K**), and *Trpa1* (**L**). **M** Schematic of assay to verify changes of β-endorphin levels in MC903-treated mice. Levels of β-endorphin in the skin (**N**) and serum (**O**) of control and MC903-treated mice. *p < 0.05, ***p < 0.001, ns = not significant

To determine whether OPRM1 in sensory neurons is critical for transmitting opioid-induced itch signals in mice, DAMGO was administered intradermally to wildtype mice, and scratching behavior was monitored (Fig. 6A). As shown in Fig. 6B, DAMGO injection elicited a robust scratching response, indicating that peripheral OPRM1 mediates opioid-induced itch in mice.

To investigate the relationship between peripheral OPRM1 and itch in detail, a disease mouse model with enhanced itch symptoms was employed. Elevated serum levels of β-endorphin—an endogenous OPRM1 agonist—have been reported in AD patients [25, 26], suggesting a link between opioid signaling and AD. Accordingly, the MC903 mouse model, a well-established model of AD [27], was used to examine OPRM1/MRGPRX1 function (Fig. 6C). MC903-treated mice displayed significantly increased spontaneous scratching compared to controls (Fig. 6D), confirming induction of an AD-like phenotype. Furthermore, primary cultures of DRG neurons from MC903-treated mice exhibited heightened calcium responses to DAMGO (Fig. 6E and 6F), indicating upregulated OPRM1 activity in sensory neurons associated with AD. Importantly, this enhanced DAMGO-evoked calcium response was markedly suppressed when *MrgprX1* was knocked down using siRNA (Fig. 6G), demonstrating that the increased peripheral opioid sensitivity in MC903-treated mice critically depends on MRGPRX1, presumably through the OPRM1/MRGPRX1 heterodimer.

Immunohistochemistry revealed stronger OPRM1 signals in DRG neurons from MC903-treated mice compared to controls (Fig. 6H). In contrast, MRGPRX1 staining remained similar between groups, indicating minimal expression changes (Fig. 6H). RT-qPCR analysis of DRG neurons following MC903 treatment demonstrated markedly increased *Oprm1* mRNA levels in MC903-treated mice relative to controls (Fig. 6I), whereas *MrgprX1* transcript levels remained unchanged (Fig. 6J). Analysis of *Trpv1* and *Trpa1* transcription—two itch-mediating ion channels expressed in NP2 and NP3 neurons—revealed no significant expression differences (Fig. 6K and 6L). These results indicate that OPRM1, but not MRGPRX1, is specifically upregulated in DRG neurons of MC903-treated mice.

Additionally, endogenous β-endorphin levels in MC903-treated mice were measured (Fig. 6M). A significant increase in β-endorphin levels was observed in both skin (Fig. 6N) and serum (Fig. 6O). These findings suggest that OPRM1 overexpression in sensory neurons and elevated β-endorphin levels together may contribute to the enhanced scratching behavior observed in this AD model.

### OPRD1 couples with MRGPRX2 to elicit mild calcium changes in HEK293T cells

Previous reports indicate that morphine can induce mast cell degranulation [28], thereby releasing pruritogens such as histamine and tryptase. Additionally, MRGPRX2, rather than MRGPRX1, is specifically expressed in mast cells [29]. Therefore, potential heterodimer formation between MRGPRX2 and the opioid receptors OPRM1 or OPRD1 was investigated (Fig. S3A).

Co-expression of hOPRM1 and hMRGPRX2 in HEK293T cells did not yield a detectable increase in intracellular calcium following DAMGO application (Fig. S3B), indicating that OPRM1 and MRGPRX2 do not couple to induce calcium influx. The investigation was then extended to OPRD1, another Gα_i/o_-coupled GPCR, to assess whether its co-expression with MRGPRX2 could similarly trigger calcium mobilization, as observed for OPRM1/MRGPRX1. Treatment of HEK293T cells co-expressing hOPRD1 and hMRGPRX2 (“hOPRD1/hMRGPRX2” cells) with 1 µM DPDPE—a selective OPRD1 agonist—elicited a modest increase in intracellular calcium, whereas cells expressing hMRGPRX2 alone showed no change (Fig. S3C). To assess receptor specificity, 10 µM DPDPE was applied to both hOPRM1/hMRGPRX2 and hOPRD1/hMRGPRX2 cells (Fig. S3D). A calcium increase occurred only in hOPRD1/hMRGPRX2 cells, indicating selective coupling of OPRD1 to MRGPRX2. Leucine-enkephalin (Leu-Enk)—an endogenous OPRD1 agonist—was then tested in hOPRD1/hMRGPRX2 cells. Treatment with 10 µM Leu-Enk induced a small calcium influx only in hOPRD1/hMRGPRX2 cells, with no effect in hMRGPRX2-only cells (Fig. S3E).

To confirm involvement of OPRD1 and MRGPRX2 (and its mouse ortholog MrgprB2), cells were pretreated with naltrexone—also an OPRD1 antagonist—or QWF, an MRGPRX2/MrgprB2 antagonist, prior to Leu-Enk treatment (Fig. S3F). QWF pretreatment suppressed calcium elevations induced by Leu-Enk in both hOPRD1/hMRGPRX2 and mOprd1/mMrgprB2 cells (Fig. S3G and S3H). Furthermore, naltrexone pretreatment reduced Leu-Enk-evoked calcium increases in hOPRD1/hMRGPRX2 (Fig. S3I) and mOprd1/mMrgprB2 (Fig. S3J) cells. These results indicate that OPRD1 functionally couples with MRGPRX2 to induce mild intracellular calcium changes when co-expressed in HEK293T cells.

### OPRD1 and MRGPRX2 in mast cells do not induce degranulation

Following confirmation of OPRD1/MRGPRX2 coupling in HEK293T cells, its role in mast cell degranulation was assessed (Fig. S4A). HMC-1.2 human mast cells were subjected to a β-hexosaminidase assay to measure degranulation. In HMC-1.2 cells, neither DPDPE nor Leu-Enk induced detectable degranulation (Fig. S4B and S4C). In contrast, compound 48/80—an MRGPRX2 agonist—triggered significant granule release as a positive control. DAMGO likewise failed to induce degranulation in HMC-1.2 cells (data not shown), indicating unlikely OPRM1 involvement. Therefore, although DPDPE and Leu-Enk can activate OPRD1/MRGPRX2 in HEK293T cells, they were unable to induce degranulation in HMC-1.2 cells. In addition, primary mouse peritoneal mast cells (PMCs) produced similar results. Neither DPDPE nor Leu-Enk elicited degranulation (Fig. S4D and S4E), suggesting minimal contribution of OPRD1/MrgprB2.

These findings indicate that, unlike OPRM1/MRGPRX1 coupling in sensory neurons, OPRD1/MRGPRX2 heterodimers do not trigger mast cell degranulation in human or mouse models, suggesting a minimal role in opioid-induced peripheral itch.

## Discussion

The present study identifies the OPRM1/MRGPRX1 heterodimer as a key mediator of opioid-induced itch signaling in peripheral sensory neurons, revealing a previously unrecognized function for this receptor complex. The heterodimer enables opioid-induced intracellular calcium increase, which contributes to the generation of itch signals independent of central nervous system pathways. These findings underscore the importance of peripheral receptor interactions in the pathogenesis of opioid-induced pruritus and open new avenues for therapeutic intervention.

The ability of OPRM1 to form heterodimers is well established [14]. Previous studies have demonstrated its ability to heteromerize with OPRD1 [30], as well as with other GPCRs such as V1bR [31], and Gal1R [32]. Notably, a prior study has already reported heterodimer formation between OPRM1 and MRGPRX1 [15], but this was linked primarily to psychological effects such as withdrawal, tolerance, and addiction [14]. In contrast, our findings provide functional evidence for the role of OPRM1/MRGPRX1 heterodimers in peripheral itch transmission.

Opioid-induced itch has traditionally been categorized into central and peripheral mechanisms [33, 34]. Central mechanisms, particularly those involving disinhibition in the spinal cord, have received the most attention. For instance, intrathecal administration of morphine or DAMGO induces scratching behavior in mice, while deletion of *Oprm1* in spinal inhibitory interneurons abolishes this response [7]. Despite these findings, the peripheral contribution remains underexplored.

Our data reveal a peripheral mechanism whereby opioids activate OPRM1/MRGPRX1 heterodimers in sensory neurons, producing itch signals distinct from those generated centrally. This peripheral mechanism provides a possible explanation for the paradox of opioids producing both analgesia and pruritus. Specifically, activation of OPRM1 alone suppresses pain via Gα_i/o_-coupled cAMP reduction, whereas activation of the OPRM1/MRGPRX1 heterodimer triggers Gα_q/11_-mediated calcium increase and pruritic signaling. This dual functionality suggests that selectively targeting peripheral MRGPRX1 may alleviate itch without compromising analgesic effects.

Historically, peripheral opioid-induced itch has been attributed to mast cell degranulation and subsequent histamine release [33, 35]. Morphine, for instance, has been shown to trigger degranulation and elicit wheal-and-flare reactions, as demonstrated in studies using human mast cell lines and skin testing [36, 37]. However, proteomic analyses of human mast cells reveal that canonical opioid receptors are not significantly expressed [38]. Instead, MRGPRX2 appears to play a critical role in opioid-induced mast cell degranulation, as morphine can directly activate MRGPRX2, which may evoke degranulation independently of OPRM1 [39]. Although MRGPRX2 can functionally couple to OPRD1 (Fig. S3), activation of the OPRD1/MRGPRX2 complex seems insufficient to induce mast cell degranulation (Fig. S4), indicating a limited role for opioid receptors in mast cells. Supporting this, naloxone did not inhibit morphine-induced histamine release in human skin [40]. These findings suggest that peripheral opioid-induced itch is not strongly mediated by mast cell–dependent mechanisms.

If peripheral opioid-induced pruritus is minimally associated with mast cells, the limited efficacy of antihistamines in its alleviation [41] becomes understandable, suggesting the involvement of a mast cell–independent mechanism. This is further supported by evidence showing that DAMGO induces itch even in mast cell–deficient mice [10], and that several opioids (e.g., fentanyl, sufentanil) do not significantly trigger mast cell degranulation at clinically relevant concentrations [39, 42]. Consistent with these findings, our data show that DAMGO neither activates MRGPRX2 nor induces mast cell degranulation (Fig. S3B and S4B). Therefore, DAMGO-induced scratching is less likely to result from mast cell activity but rather implies stimulation of the OPRM1/MRGPRX1 heterodimer in sensory neurons.

Clinically, opioid-induced pruritus occurs predominantly with neuraxial administration (intrathecal or epidural), being more common with intrathecal delivery than with epidural, and less frequent with oral administration [43]. Because OPRM1 is highly expressed in the spinal cord, central mechanisms have traditionally been considered the primary drivers of opioid-induced itch. However, as itch is fundamentally a peripheral sensation and difficult to dissociate from peripheral mechanisms, and because mast cell degranulation does not fully explain peripheral opioid-induced pruritus, the involvement of additional peripheral pathways cannot be excluded. Our findings demonstrate that opioid-induced pruritus can be mediated by the OPRM1/MRGPRX1 interaction in sensory neurons, highlighting a peripheral pathway that has been largely overlooked.

It remains unclear whether intrathecally administered opioids induce pruritus solely through central mechanisms or also involve peripheral pathways. However, intrathecal opioids have access to both spinal cord and DRG, which are bathed in cerebrospinal fluid (CSF) [44]. Research has shown that even large molecules such as antisense oligonucleotides and viral vectors can reach DRG neurons through intrathecal delivery [45, 46]. Moreover, intrathecal administration of opioids such as morphine can suppress excitability and nociceptive transmission in DRG neurons [44]. For example, studies have demonstrated that intrathecal injections can stain both DRG and spinal nerves [47], while other studies confirm partial extension of CSF into the DRG [48, 49]. This suggests that neuraxially administered opioids may also exert peripheral effects that contribute to pruritus, although this possibility warrants further research to determine the underlying pathways.

Additionally, sensory neurons from MC903-treated AD model mice exhibited enhanced responses to DAMGO (Fig. 6E and 6F), suggesting a connection between opioid signaling and AD-related itch. These mice also showed elevated OPRM1 expression (Fig. 6H and 6I) and increased β-endorphin levels (Fig. 6N and 6O), indicating heightened peripheral sensitivity to endogenous opioids that may amplify itch signaling. Indeed, β-endorphin, along with IgE, is recognized as a biomarker for itch and disease severity in AD [50, 51]. Furthermore, upregulation of genes encoding the β-endorphin precursor (*POMC*) and *OPRM1* has been reported in the skin of AD patients [52, 53], and dysregulated OPRM1 signaling may contribute to the pathogenesis of pruritic conditions [9]. On the other hand, although we cannot entirely exclude the possibility that BAM8-22 may be altered in AD, its involvement remains unclear, and no evidence currently indicates elevated BAM8-22 levels in AD skin. Taken together, these observations point toward β-endorphin, rather than BAM8-22, as a key contributor to opioid-related itch in AD, at least under our experimental conditions. These findings suggest that peripheral OPRM1 overexpression may underlie a significant role for AD-associated itch. Further studies should explore how increased OPRM1 in sensory neurons, along with its interaction with MRGPRX1, contributes to the development and severity of itch in AD.

While this study highlights the unexpected role of the OPRM1/MRGPRX1 heterodimer in itch transmission, several limitations must be acknowledged. Although our data support a functional interaction between OPRM1 and MRGPRX1, the absence of heterodimer-selective antagonists or neutralizing antibodies currently limits the ability to directly and selectively interrogate the OPRM1/MRGPRX1 heterodimer. The development of such selective reagents in the future would allow more definitive mechanistic studies. Another limitation is that our in vivo experiments relied on mouse MrgprX1, which, although functionally orthologous to human MRGPRX1, does not permit direct assessment of the interaction between human OPRM1 and human MRGPRX1. In addition, although the OPRM1/MRGPRX1 heterodimer does not alter the G-protein coupling preferences of either receptor, other downstream signaling pathways or receptor interactions that may influence opioid-induced itch were not investigated. A more comprehensive analysis of such downstream events would further clarify the mechanisms by which this heterodimer contributes to pruritic signaling. Furthermore, most experiments were conducted using in vitro systems and animal models, which, while useful for elucidating mechanisms, do not perfectly mirror human physiology. Further research is needed to determine whether the OPRM1/MRGPRX1 heterodimer functions similarly in humans and contributes to opioid-induced pruritus in clinical settings.

Finally, although this study focuses on OPRM1, it does not exclude the potential roles of other opioid receptors in itch modulation. For instance, OPRD1 has been shown to regulate itch bidirectionally [54], and OPRK1 agonists such as difelikefalin and nalfurafine have shown promise as antipruritic agents [55]. Nevertheless, our findings emphasize the novel linkage between OPRM1 and MRGPRX1 in peripheral sensory neurons. This newly identified interaction not only advances our understanding of opioid-induced pruritus but also highlights potential therapeutic strategies aimed at reducing itch without compromising analgesic efficacy.

## Conclusion

The present study reveals a previously unrecognized peripheral mechanism of opioid-induced pruritus mediated by OPRM1/MRGPRX1 heterodimers in sensory neurons. This receptor complex facilitates opioid-induced calcium increase in sensory neurons, thereby initiating peripheral itch signaling. Our results underscore the significance of peripheral receptor crosstalk in the pathogenesis of opioid-induced itch and suggest a novel mechanistic basis distinct from traditional central or mast cell–mediated pathways. Targeting this heterodimer may offer a promising therapeutic strategy to mitigate opioid-induced itch without diminishing analgesic efficacy. Further investigation is warranted to assess the clinical relevance of this mechanism and to explore whether additional opioid receptor interactions contribute to this multifaceted response.

## Supplementary Information

Supplementary material 1.

## Data Availability

The datasets used and analyzed during the current study are available from the corresponding author upon reasonable request.

## Abbreviations

AD: Atopic dermatitis
BAM8-22: Bovine adrenal medulla 8–22
BiFC: Bimolecular fluorescence complementation
cAMP: Cyclic adenosine monophosphate
DAG: Diacylglycerol
DMEM: Dulbecco’s Modified Eagle Medium
DRG: Dorsal root ganglion
ELISA: Enzyme-linked immunosorbent assay
FBS: Fetal bovine serum
Gα_i/o_: G protein alpha subunit i/o type
Gα_q/11_: G protein alpha subunit q/11 type
GPCR: G Protein–Coupled Receptor
HEK293T: Human Embryonic Kidney 293T cells
IP_3_: Inositol 1,4,5-Trisphosphate
MC903: Calcipotriol
MRGPRX1: Mas-Related G Protein–Coupled Receptor X1
MRGPRX2: Mas-Related G Protein–Coupled Receptor X2
OPRD1: δ-Opioid receptor
OPRK1: κ-Opioid receptor
OPRM1: μ-Opioid receptor
ORL-1: Opioid receptor-like 1
PBS: Phosphate-buffered saline
PIP_2_: Phosphatidylinositol 4,5-bisphosphate
PLC: Phospholipase C
PMC: Peritoneal mast cell
PTX: Pertussis toxin
RT-qPCR: Real-time quantitative polymerase chain reaction
siRNA: Small interfering RNA
VN/VC: Venus N-terminal/C-terminal fragment
