# Supplementary material for: OPRM1/MRGPRX1 heterodimers drive opioid-induced itch through a peripheral mechanism
Source: J Biomed Sci. 2026 Mar 28;33:35. doi: 10.1186/s12929-026-01238-x (PMC13032443; doi:10.1186/s12929-026-01238-x)
Supplement: Supplementary file 1 — Supplementary material 1. [file 12929_2026_1238_MOESM1_ESM.docx]

Supplementary Figures


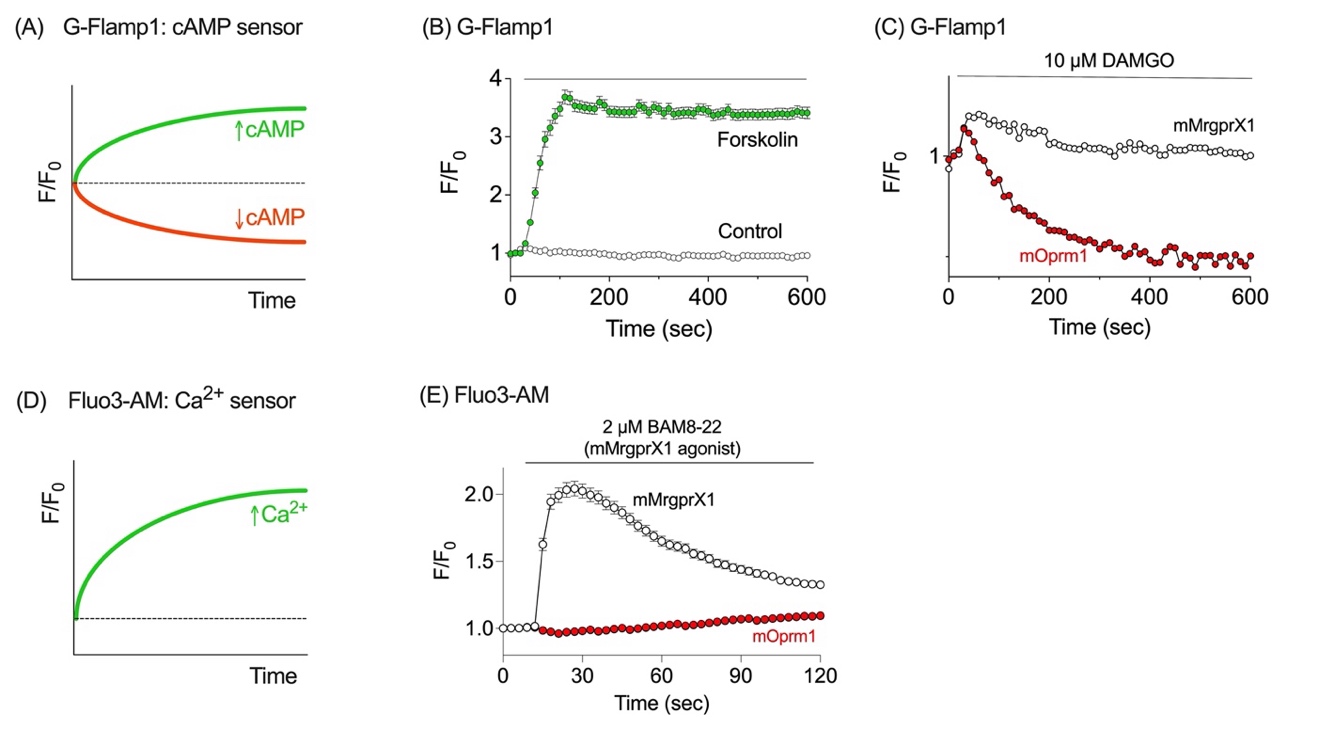


Supplementary Figure 1. cAMP and calcium signaling dynamics in cells expressing mOprm1 and mMrgprX1.

(A) Schematic representation of the G-Flamp1 sensor used to measure cAMP levels. Increases in cAMP lead to fluorescence intensity changes, with upward or downward trends depending on activation. (B) Time-course fluorescence changes (F/F₀) in HEK293T cells expressing G-Flamp1 treated with forskolin (a cAMP activator) or control, demonstrating that the sensor responds to cAMP. (C) DAMGO (10 µM)-induced cAMP signaling in HEK293T cells expressing mOprm1 or mMrgprX1. (D) Schematic representation of the Fluo3-AM calcium sensor used to measure intracellular calcium mobilization. (E) Time-course fluorescence changes (F/F₀) in cells treated with BAM8-22 (2 µM, mMrgprX1 agonist). Calcium responses are observed only in cells expressing mMrgprX1, but not in mOprm1 cells.


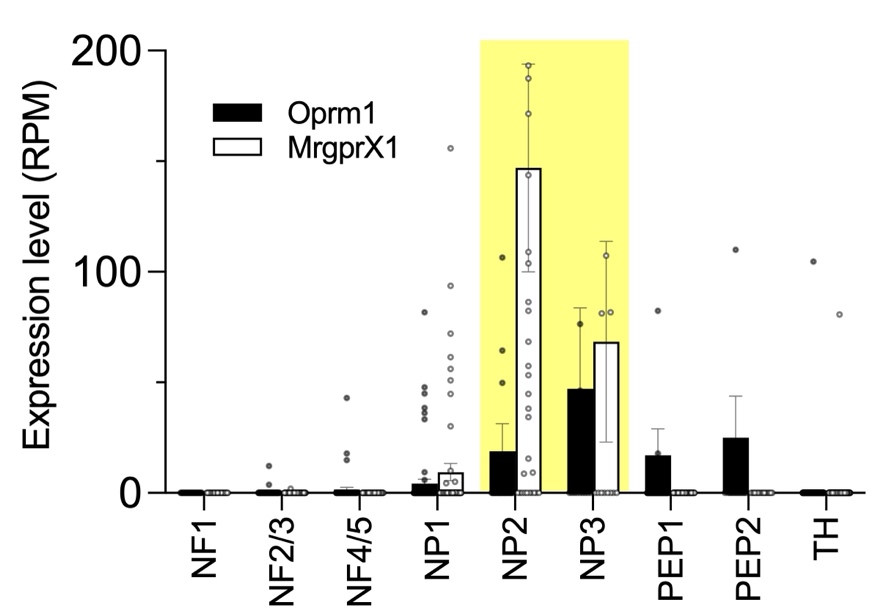


**Supplementary Figure 2. Co-expression of *Oprm1* and *MrgprX1* in specific mouse DRG neuron subtypes.**

RNA sequencing analysis showing expression patterns of *Oprm1* and *Mrgprx1* across DRG neuronal subtypes. Notable co-expression of *Oprm1* and *Mrgprx1* is observed in NP2 and NP3 populations. RNA sequencing data were retrieved from the Gene Expression Omnibus (GEO) under accession number GSE59739. Original data were described in the article by Usoskin et al.

**
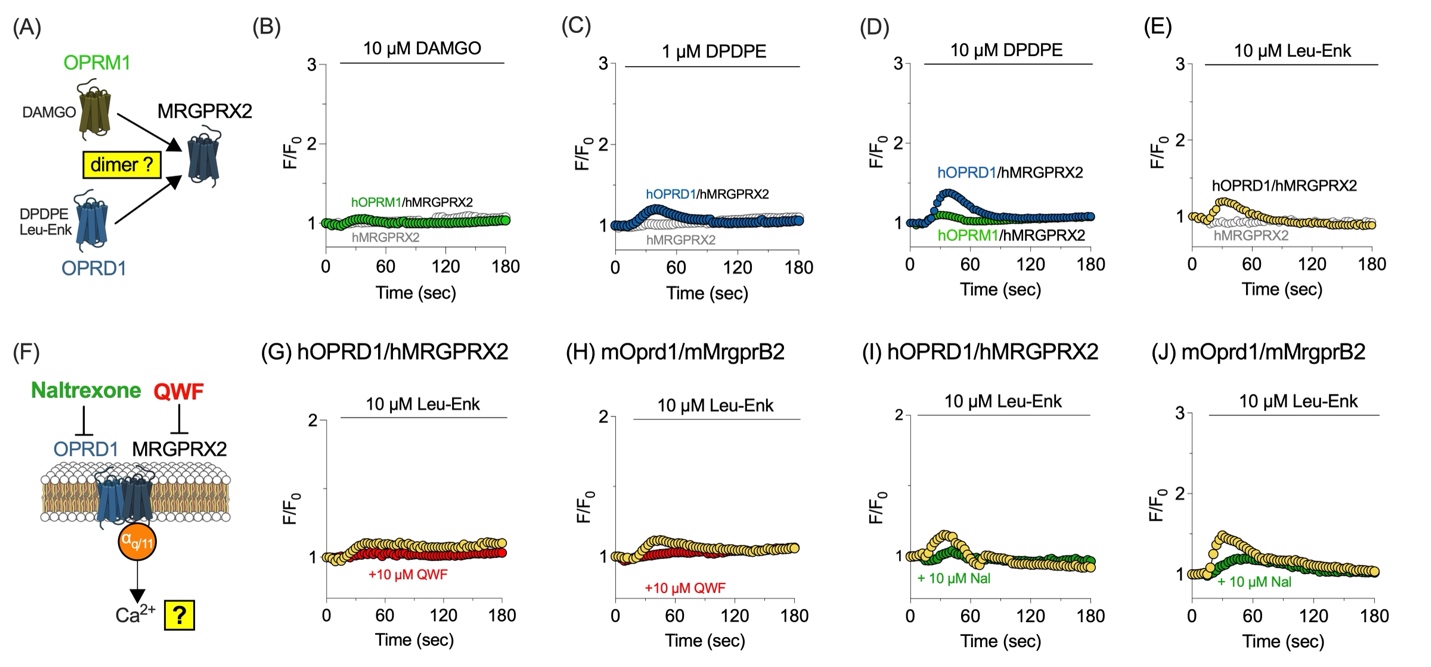
**

**Supplementary Figure 3. Coupling of OPRD1 with MRGPRX2 induces mild calcium mobilization**

(A) Schematic of potential heterodimer interactions among OPRM1, OPRD1 and MRGPRX2, tested by calcium imaging. (B) Time course of normalized fluorescence (F/F_0_) in HEK293T cells co-expressing hOPRM1 and hMRGPRX2 following 10 µM DAMGO, showing no detectable calcium response. (C) Time course of F/F_0_ in HEK293T cells co-expressing hOPRD1 and hMRGPRX2 after treatment with 1 µM DPDPE, revealing a modest calcium increase. (D) Comparison of calcium responses in hOPRD1/hMRGPRX2 and hOPRM1/hMRGPRX2 cells treated with 10 µM DPDPE. (E) Time course of F/F_0_ in hOPRD1/hMRGPRX2 cells treated with 10 µM Leu-enkephalin (Leu-Enk). (F) Schematic of inhibitor assays verifying blockade of OPRD1 or MRGPRX2 prior to Leu-Enk treatment. (G, H) Time courses of Leu-Enk–induced F/F_0_ in hOPRD1/hMRGPRX2 (G) and mOprd1/mMrgprB2 (H) cells with or without 10 µM QWF. (I, J) Time courses of Leu-Enk–induced F/F_0_ in hOPRD1/hMRGPRX2 (I) and mOprd1/mMrgprB2 (J) cells with or without 10 µM naltrexone.

**
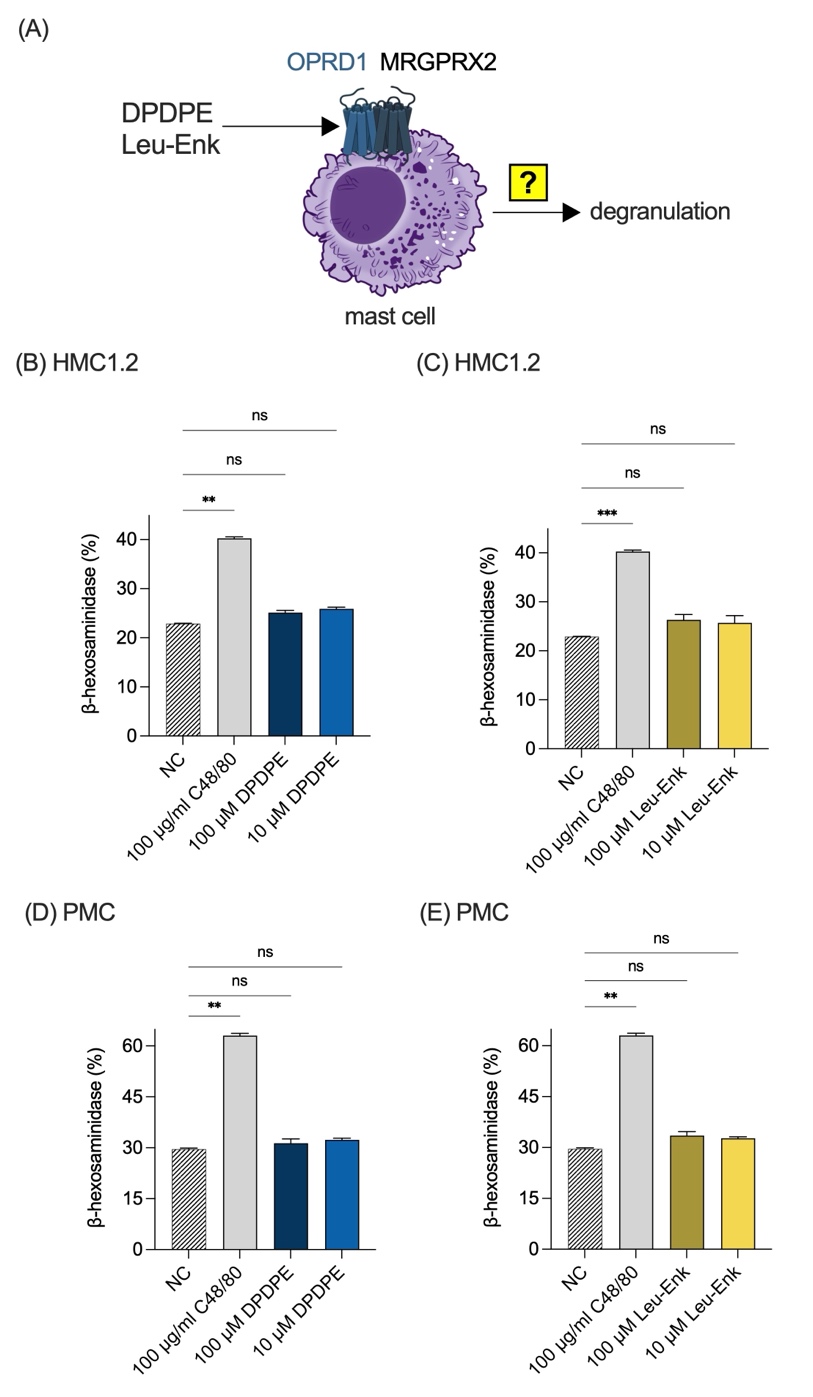
**

**Supplementary Figure 4. OPRD1 and MRGPRX2 coupling does not induce mast cell degranulation**

(A) Schematic of mast cell degranulation assay in mast cells. Degranulation was assessed following stimulation with DPDPE or Leu-enkephalin (Leu-Enk). (B, C) β-hexosaminidase release (%) in HMC1.2 cells treated with NC (negative control), C48/80 (100 µg/mL, MRGPRX2 agonist), DPDPE (10 µM and 100 µM, an OPRD1 agonist), or Leu-Enk (10 µM and 100 µM, an OPRD1 agonist). (D, E) β-hexosaminidase release (%) in mouse peritoneal mast cells (PMCs). **p < 0.01, ***p < 0.001, ns = not significant.
